# EEG-Neurofeedback in psychodynamic treatment of substance dependence

**DOI:** 10.3389/fpsyg.2013.00692

**Published:** 2013-10-01

**Authors:** Human F. Unterrainer, Andrew J. Lewis, John H. Gruzelier

**Affiliations:** ^1^Centre for Integrative Addiction Research (Grüner Kreis Society)Vienna, Austria; ^2^Department of Psychology, Karl-Franzens-UniversityGraz, Austria; ^3^Faculty of Health, School of Psychology, Deakin UniversityMelbourne, VIC, Australia; ^4^Department of Psychology, Goldsmiths, University of LondonLondon, UK

**Keywords:** neuropsychoanalysis, neurofeedback, psychodynamic psychotherapy, addiction, anhedonia, theta, SMR

The relationship between therapeutic techniques and psychoanalytic theory is complex and defies a direct translation from theory to practice. However, especially in recent years, there have been increased efforts to bolster psychodynamic research by drawing on neuroscientific findings. Proponents of this approach argue that the so-called “neuropsychoanalytic” models are only drawing upon Freud's own originally neurological framework- reflecting one of the central disciplinary origins of psychoanalysis (Kaplan-Solms and Solms, 2000). Indeed, Freud considered his psychologically based models a temporary structure which would later be reinforced once mechanistic neurological processes were identified which corresponded to the psychological processes he described. While not entering the specifics of this claim, it is reasonable to suggest that the brain-mind relationship has always been fundamental to psychoanalysis but this relationship has not been exploited within its clinical techniques.

One means of further exploring the real time relationship between psychodynamic clinical processes and their manifestation in neuroprocessing is the integration of neurofeedback into psychodynamic sessions. Practically speaking, both interventions could be integrated, or at least inform one another. How this could be done is yet to be established.

We would recommend that the interventions are done in parallel, for the neurofeedback protocols address cognitive dysfunction sensory-motor rhythm (SMR) and well-being (A/T) so that more is gained from the psychotherapy which is necessary from the outset to provide psychodynamic support. For logistical reasons all three were done in an afternoon in our study but with hindsight this proved too taxing and separate sessions for all three would be recommended. Neurofeedback (NF) can be characterized as a form of instrumental learning using feedback in real time from the brain's electrical activity. Participants learn to regulate their brain activity themselves through a process of repetition and reinforcement (Gruzelier, 2012). NF training has been shown to be an effective tool to aid in the treatment of a wide range of psychiatric disorders. For one example, a substantial body of research has been conducted over the past three decades by Peniston using a slow EEG-wave protocol for the treatment of addictive disorders (Peniston and Kulkovsky, 1999). This “Peniston-Protocol” became very popular and widely accepted as a research paradigm and has shown to be effective in a number of studies (Scott et al., 2005; Sokhadze et al., 2008). Substance use disorders result in specific alteration in brain activity that is detectable with the use of quantitative electroencephalography (EEG) methods (Peled, 2008). Psychodynamic psychotherapy aims to identify and modify these enduring patterns of thoughts, feelings, impulses, and defenses that, in turn, lead to maladaptive compromises, ineffective behavior, and conflicts in interpersonal relations (Shafranske, 2009). So the therapeutic question is in which way the altered brain activity can be normalized by either NF, psychodynamic therapy or the combination?

To further illustrate this we refer here to very limited data coming from a single case treatment of a student who, on a weekly basis throughout a university term, was given short-term psychodynamic psychotherapy and two EEG-neurofeedback protocols: SMR and alpha/theta (A/T) (Unterrainer et al., 2013). Substance misuse produces a diversity of EEG irregularities; there is no characteristic pattern. Accordingly neurofeedback therapy has focused on remedying the cognitive and affective deficits such as attention, impulsivity, anhedonia, etc. Our case study approach was informed by Scott et al., (2005) who preceded A/T training with the a course of SMR training (see Egner and Gruzelier, 2001) reporting a beneficial outcome from the combined neurofeedback protocols on impulsive errors and reaction-time variability in a sustained attention task, aside from a reduction in alienation, depression and defensiveness. Here we attempted to treat a long term drug misuse habit co-occurring with a depressive mood disorder (Lewis et al., 2008). In this single case psychodynamic psychotherapy sessions were always applied right before the Neurofeedback training on the same day. As a result the patient's capacity for attention as evinced by application to course work in English literature, improved substantially, and there was a striking reduction in psychopathology. The improvement was much more rapid than we would have anticipated from either therapy alone. This tentative finding suggests a number of avenues for further exploration. For example, given the increased interest in the neurobiological foundations of psychoanalytic theory, EEG-neurofeedback could potentially also be used to understand therapeutic change processes (Linden, 2006). Exactly how that would be achieved, would require a repeating combined NF/ psychodynamic treatment approach in order to observe, how the NF learning outcome differs between positive and negative therapy outcomes. In order to confirm the interaction effect of these combined therapeutic interventions a randomized controlled trial would be necessary. Another recommendation would be to schedule the psychotherapy and neurofeedback sessions on separate days and also schedule the SMR and A/T protocols on different days. There are studies from other groups which have tried to combine neurofeedback and psychotherapy (Arns et al., 2009), however, to our knowledge this case is the first one, in which Neurofeedback was related to a strictly psychodynamic approach and certainly the first for drug misuse. Hopefully future studies will confirm our initial impression in order to further develop a NF informed model of psychodynamic psychotherapy, and to apply this beyond cases of substance misuse.
